# Association of daily tar and nicotine intake with incident myocardial infarction: Results from the population-based MONICA/KORA Augsburg Cohort Study 1984 - 2002

**DOI:** 10.1186/1471-2458-11-273

**Published:** 2011-05-04

**Authors:** Qiu-Li Zhang, Jens Baumert, Karl-Heinz Ladwig, H-Erich Wichmann, Christa Meisinger, Angela Döring

**Affiliations:** 1 Institute of Epidemiology II, Helmholtz Zentrum München, German Research Center for Environmental Health, Ingolstädter Landstrasse 1, 85764 Neuherberg, Germany; 2 Institute of Epidemiology I, Helmholtz Zentrum München, German Research Center for Environmental Health, Ingolstädter Landstrasse 1, 85764 Neuherberg, Germany; 3 Institute of Medical Informatics, Biometry and Epidemiology (IBE), Ludwig-Maximilians-Universität München, Marchioninistr. 15, 81377 München, Germany

## Abstract

**Background:**

Cigarette smoking has been shown to be one of the most important risk factors for cardiovascular diseases. However, little is known about cumulative effects of daily tar and nicotine intake on the risk of incident myocardial infarction (MI) so far. To bridge this gap, we conducted an analysis in a large prospective study from Southern Germany investigating associations of daily tar and nicotine intake with an incident MI event.

**Methods:**

The study was based on 4,099 men and 4,197 women participating in two population-based MONICA Augsburg surveys between 1984 and 1990 and followed up within the KORA framework until 2002. During a mean follow-up of 13.3 years, a number of 307 men and 80 women developed an incident MI event. Relative risks were calculated as hazard ratios (HRs) estimated by Cox proportional hazards models adjusted for cardiovascular risk factors.

**Results:**

In the present study, male regular smokers consumed on average more cigarettes per day than female regular smokers (20 versus 15) and had a higher tar and nicotine intake per day. In men, the MI risk compared to never-smokers increased with higher tar intake: HRs were 2.24 (95% CI 1.40-3.56) for 1-129 mg/day, 2.12 (95% CI 1.37-3.29) for 130-259 mg/day and 3.01 (95% CI 2.08-4.36) for ≥ 260 mg/day. In women, the corresponding associations were comparable but more pronounced for high tar intake (HR 4.67, 95% CI 1.76-12.40). Similar associations were observed for nicotine intake.

**Conclusions:**

The present study based on a large population-based sample adds important evidence of cumulative effects of tar and nicotine intake on the risk of incident MI. Even low or medium tar and nicotine intake revealed substantial risk increases as compared to never-smokers. Therefore, reduction of tar and nicotine contents in cigarettes cannot be seen as a suitable public health policy in preventing myocardial infarction.

## Background

Cigarette smoking is a central issue in public health policy as it has been shown to be associated with an elevated risk of various cardiovascular diseases and types of cancer [1,2]. Smoking has been determined as one of the most important risk factors for myocardial infarction (MI) [3-5], but it was shown that smoking cessation can reduce this risk [6]. Many countries and international agencies have made great efforts to change smoking behaviour and to encourage smokers to quit smoking, e.g. by preventing initiation of tobacco use, promoting cessation among adolescents and adults, or banning advertising and promotions [7]. The need for regulation and legislation on limits of harmful substances, including tar and nicotine content in cigarettes, has recently drawn a lot of attention. The European Union recommended that the upper limit of tar content should be decreased from 12 mg to 10 mg. Such regulatory efforts might modify cigarette design in tobacco industry and might be seen as a further focus in smoking preventing policy, although it as been shown that even so-called "light" cigarettes with reduced tar and nicotine yield might have adverse effects on the health status.

However, little is known on how lower tar and nicotine contents in cigarettes can change overall smoking behaviour and subsequently have an affect on the risk for smoking-related diseases like MI. The associations between the number of smoked cigarettes and quantity of tar and nicotine yield of smoked cigarette remains unclear. Several studies have investigated effects of low yield cigarette by reductions of tar and nicotine emissions on the MI risk, however results are inconclusive [8-12].

To bridge this gap, the present analysis based on a population-based prospective study was carried out to investigate the extent of cumulative effects of daily tar and nicotine intake on the risk of an incident fatal or non-fatal MI event including coronary death. We would like to give an answer to the question whether a reduction of tar and nicotine contents in cigarettes might be seen as a suitable public health policy in preventing the incidence of a MI. Due to sex-specific differences in smoking behaviour and MI incidence, we conduct all analyses separately in men and women.

## Methods

### Study design and study population

The present study was derived from the population-based MONICA (Monitoring Trends and Determinants in Cardiovascular Diseases)/KORA (Cooperative Health Research in the Region of Augsburg) Augsburg surveys conducted between 1984 and 1995 [5,13]. The World Health Organization (WHO) MONICA Project was initiated in the early 1980s in 26 countries to monitor the risk factors for cardiovascular diseases, such as hypertension, smoking, hypercholesterolemia and obesity [14]. The MONICA Augsburg Study, a part of the multinational WHO MONICA project, was initiated in 1984 in the city of Augsburg and two adjacent counties in southern Germany. The study was approved by the local authorities: The MONICA surveys S1 and S2 with the baseline examination were approved by the data protection commission following the rules at the time of the examinations (1984/85 and 1989/90). The follow-up examinations within the KORA framework were approved by the ethics committee of the Bavarian Medical Association. All participants provided a written informed consent.

Overall, a number of 8,802 persons participated in the first survey (S1) conducted in 1984/85 (age range 25 to 64 years, response 79*%*) or in the second survey (S2) conducted in 1989/90 (age range 25 to 74 years, response 77*%*). After excluding 506 participants with a history of MI or with incomplete information on any of the considered variables, the study population of the present analysis comprised of 8,296 subjects (4,099 men and 4,197 women).

Baseline information on socio-demographical and lifestyle characteristics as well as medical examinations including collection of a nonfasting venous blood sample was assessed by trained medical staff in a standardized manner following the WHO MONICA recommendations [15].

### Definition of incident MI

Within the framework of KORA study participants were followed up until 2002. The outcome incident MI was defined as the first event of a non-fatal or fatal MI including coronary death before the age of 75 years and was assessed by the MONICA/KORA Augsburg coronary event registry [16]. Until December 2000, the diagnosis of a major non-fatal MI event was based on the MONICA algorithm taking into account symptoms, cardiac enzymes and electrocardiography (ECG) changes. Since January 1, 2001 MI was diagnosed according to ESC and ACC criteria. Vital status of all cohort members was assessed regularly through the population registries. Deaths from MI were validated by autopsy reports, death certificates, chart review, and information from the last treating physician.

Mean duration of follow-up was 13.3 years (standard deviation (SD) 4.4) and ranged from 0.03 to 18.2 years. During the follow-up period, a number of 307 men and 80 women developed an incident MI event.

### Definition of smoking status, tar and nicotine intake

Smoking status was assessed by a face-to-face interview by asking "Do you currently smoke cigarettes?". In case of answering "yes", participants were further asked if they smoke regularly or occasionally; in case of answering "no", participants were asked "Have you ever smoked cigarettes?". Moreover, the average amount of cigarettes smoked per day among regular smokers was assessed in the questionnaire by the question "How many cigarettes do you smoke on average per day?" and age at smoking onset by the question "How old have you been when you started to smoke cigarettes?".

Participants were classified into four categories: regular smokers, occasional smokers, ex-smokers and never-smokers. Regular smokers are defined as those who reported to smoke currently at least one cigarette per day; while occasional smokers smoked less than one cigarette per day on average. Ex-smokers are those subjects who smoked cigarettes daily before the time of baseline examination but not currently. Never-smokers reported to have not consumed any cigarettes before the time of baseline examination. The number of cigarettes smoked on average per day (cig/day) was divided into light smokers (1-19 cig/day) and heavy smokers (≥ 20 cig/day).

Information on tar and nicotine contents of cigarette for each brand was obtained from annual reports by the respective cigarette manufactures. Among the regular smokers, nicotine intake per day (mg/day) was calculated by multiplying the nicotine yield per cigarette smoked with the number of cigarettes smoked per day, and tar intake per day (mg/day) was calculated by multiplying the tar yield per cigarette smoked with the number of cigarettes smoked per day. Using tertiles of the distribution of nicotine yield per cigarette, nicotine intake per day was divided into groups of low (≤ 8 mg/day), medium (9 - 16 mg/day), and high nicotine intake per day (≥ 17 mg/day) among regular smokers. Using tertiles of the distribution of tar yield per cigarette, tar intake per day then was grouped into groups of low (≤129 mg/day), medium (130 - 259 mg/day), and high tar intake per day (≥ 260 mg/day) among regular smokers.

### Definition of cardiovascular risk factors

Venipuncture was performed on the sitting subjects with minimal tourniquet use. Further blood handling followed strict standardization. Total serum cholesterol and high density lipoprotein cholesterol (HDL-C) were measured by enzymatic methods (CHOD-PAP, Boehringer Mannheim, Germany). HDL-C was precipitated with phosphotungstic acid and magnesium ions. For the present analyses, we used the ratio of total cholesterol and HDL-C (total cholesterol/HDL-C). Alcohol intake was assessed by a recall method and alcohol consumption was calculated in grams/day (g/day). Alcohol consumption was classified into three categories: non-drinkers (0 g/d), intake of 0.1-39.9 g/day and ≥ 40.0 g/day for men and intake of 0.1-19.9 g/day and ≥ 20.0 g/day for women. To assess physical activity, participants were considered as active during leisure time if they regularly participated in sports in summer and in winter and if they were active for at least one hour per week in either season. All other participants were considered as inactive. Actual hypertension was defined as blood pressure values ≥ 140/90 mmHg and/or use antihypertensive medication, given that the subjects were aware of being hypertensive. Diabetes was defined if participants reported a history of diabetes or if they reported use of anti-diabetic medication.

### Statistical analysis

We conducted a descriptive analysis by baseline characteristics and risk factors separately for men and women to give a description of the study population. Multivariable analyses were performed by Cox proportional hazards models to assess the effect of smoking habits, tar and nicotine intake on incident MI with controlling for potential confounding by other cardiovascular risk factors. Never-smokers were chosen as reference category. All models were calculated separately for men and women. A first basic model was adjusted for age (continuous) and survey (S1 or S2); a second multivariable model was adjusted additionally for the following variables: alcohol consumption (men: 0, 1-39, ≥ 40 g/day, women: 0, 1-19, ≥ 20 g/day), actual hypertension (yes or no), ratio of total cholesterol and HDL-C (<3.0, 3.0-5.4, ≥ 5.5), physical inactivity (yes or no) and history of diabetes (yes or no). Results are presented as hazard ratio (HR) with 95% confidence interval (95% CI). Significance tests were 2-tailed. For all statistical analysis a p value less than 0.05 was considered to be statistically significant. The evaluations were performed with the statistical software package SAS (Version 9.1, SAS-Institute Inc., Cary, NC, USA).

## Results

### Description of study population

Overall, more men (30.4%) than women (17.9%) smoked regularly in the present study population, while a higher percentage of women were never-smokers compared to men (28.8% of men, 62.3% of women). Male regular smokers consumed on average 20 cigarettes per day compared to female regular smokers with a mean of 15 cigarettes per day. Moreover, the mean intake was higher in male than in female smokers both for tar (264 versus 162 mg/day) and nicotine (17 versus 11 mg/day). Therefore, among male regular smokers, the majority belonged to the high tar and nicotine intake group (52.9% and 43.1%), while female regular smokers had more frequently a low tar and nicotine intake (43.1% and 48.9%).

The distribution of baseline characteristics and risk factors according to smoking status including tar and nicotine intake per day are presented in table 1 for men and table 2 for women. The mean age at baseline examination was higher in the low tar or nicotine intake group than in the high tar or nicotine intake group. With higher tar or nicotine intake, the cardiovascular risk factors total cholesterol/HDL-C, alcohol consumption and physical inactivity increased and actual hypertension decreased in mean or proportion which was more pronounced in men than in women. In both sexes, smokers with a high tar and nicotine intake per day started to smoke at an earlier age and had a shorter duration of smoking than those with low or medium tar and nicotine intake per day.

**Table 1:** Baseline characteristics and risk factors according to smoking status including tar and nicotine intake (mg/day) among men (n = 4,099)

Characteristics	Never- smokers	Ex- smokers	Occasional smokers	Tar intake (mg/day)			Nicotine intake (mg/day)		
				Low (≤ 129)	Medium (130 - 259)	High (≥ 260)	Low (≤ 8)	Medium (9 - 16)	High (≥ 17)
No. of participants (n)	1,181	1,532	141	236	350	659	290	418	537
Mean age (years)	46.0	50.9	43.9	49.3	44.0	42.2	48.2	43.7	42.1
Mean total cholesterol/HDL-C	4.8	5.1	4.8	5.0	5.2	5.5	5.0	5.3	5.6
Mean alcohol consumption (g/day)	26.1	32.4	36.5	34.3	33.1	45.0	34.5	36.5	44.8
Physical inactivity (%)	57.1	55.1	56.0	60.6	56.3	67.7	56.9	58.4	70.2
Actual hypertension (%)	40.6	47.9	36.9	47.9	35.7	36.9	43.5	37.6	36.9
Diabetes (%)	2.5	5.2	2.8	4.7	4.3	3.2	4.1	4.1	3.4
Mean age at smoking onset (years)	-	-	18.9	19.5	18.1	17.6	19.4	18.0	17.6
Mean duration of smoking (years)	-	-	25.7	30.5	26.5	25.2	29.5	26.4	25.2

**Table 2:** Baseline characteristics and risk factors according to smoking status including tar and nicotine intake (mg/day) among women (n = 4,197)

Characteristics	Never- smokers	Ex- smokers	Occasional smokers	Tar intake (mg/day)			Nicotine intake (mg/day)		
				Low (≤ 129)	Medium (130 - 259)	High (≥ 260)	Low (≤ 8)	Medium (9 - 16)	High (≥ 17)
No. of participants (n)	2,613	693	139	324	238	190	368	235	149
Mean age (years)	50.0	44.0	39.8	42.9	40.6	38.8	42.5	40.0	39.6
Mean total cholesterol/HDL-C	4.0	3.7	3.6	3.9	4.0	4.1	4.0	4.0	4.0
Mean alcohol consumption (g/day)	8.4	11.0	12.8	10.5	12.3	13.8	10.9	12.9	12.7
Physical inactivity (%)	66.3	55.0	50.4	61.7	63.5	71.6	62.8	61.3	75.2
Actual hypertension (%)	35.5	23.2	21.6	21.3	18.9	17.9	20.9	17.5	20.1
Diabetes (%)	3.8	3.2	2.2	0.9	0.8	1.1	1.1	0.4	1.3
Mean age at smoking onset (years)	-	-	22.0	21.5	19.3	18.4	21.3	19.2	18.4
Mean duration of smoking (years)	-	-	18.4	22.1	21.8	21.0	21.9	21.4	21.9

### Risk of incident MI by smoking habits and number of cigarettes per day

The association between smoking habits and the risk of an incident MI event with two categories for regular smokers classified by the number of cigarettes smoked per day is shown in the first part of table 3 for men and of table 4 for women. Compared to never-smokers, male regular smokers had a risk-factor-adjusted HR of 2.34 (95% CI 1.60-3.43) when consuming 1-19 cigarettes per day and of 2.71 (95% CI 1.88-3.89) when consuming 20 or more cigarettes per day. In women, the respective HRs were 1.68 (95% CI 0.79-3.57) and 3.86 (95% CI 1.57-9.50) showing a strong risk increase compared to never-smokers when consuming 20 or more cigarettes per day. These effects might indicate that the risk associated with increasing numbers of cigarettes was greater in women. Risks for ex- and occasional smokers were lower in men than in women compared to regular smokers. In women, the HR for smokers consuming 1-19 cigarettes per day was higher than for ex-smokers (1.93, 95% CI 1.07-3.47) and comparable to occasional smokers (1.60, 95% 0.39-6.65).

**Table 3:** Risk of incident myocardial infarction by number of cigarettes per day (cig/day), tar and nicotine yield per cigarette (mg/cig) and tar and nicotine intake per day (mg/day) in men: Hazard ratio (HR) and 95% confidence interval (95% CI)

Smoking habits	Number of participants (N)	Number of events (N)	Age- and survey-adjusted model* HR (95% CI)	MI risk factor-adjusted model^+^ HR (95% CI)
Never-smokers^++^	1,181	53	1.00	1.00
Ex-smokers	1,532	117	1.31 (0.94-1.81)	1.26 (0.90-1.75)
Occasional smokers	141	7	1.14 (0.52-2.50)	1.15 (0.52-2.53)
Regular smokers				
Number of cigarettes smoked per day				
Light smokers (1 - 19 cig/day)	503	54	2.54 (1.74-3.71)	2.34 (1.60-3.43)
Heavy smokers (≥ 20 cig/day)	742	76	3.13 (2.20-4.45)	2.71 (1.88-3.89)

Tar yield per cigarette*^# ^*				
1 - 12 mg/cig	274	29	2.21 (1.40-3.47)	2.05 (1.30-3.24)
≥ 13 mg/cig	971	101	3.12 (2.23-4.36)	2.73 (1.94-3.84)
				
Nicotine yield per cigarette*^# ^*				
0.1 - 0.7 mg/cig	229	26	2.36 (1.48-3.78)	2.20 (1.37-3.52)
≥ 0.8 mg/cig	1,016	104	3.01 (2.16-4.19)	2.64 (1.88-3.71)

Tar intake per day*^#^*				
1 - 129 mg/day	236	27	2.36 (1.48-3.75)	2.24 (1.40-3.56)
130 - 259 mg/day	350	33	2.36 (1.53-3.65)	2.12 (1.37-3.29)
≥ 260 mg/day	659	70	3.49 (2.43-5.01)	3.01 (2.08-4.36)
				
Nicotine intake per day*^#^*				
1 - 8 mg/day	290	34	2.46 (1.60-3.78)	2.33 (1.51-3.59)
9 - 16 mg/day	418	38	2.38 (1.57-3.61)	2.11 (1.38-3.21)
≥ 17 mg/day	537	58	3.68 (2.53-5.36)	3.16 (2.15-4.65)

++ Reference category, * Model was adjusted for age (continuous) and survey (S1 or S2), model was additionally adjusted for alcohol consumption per day (0, 1-39, ≥ 40 g/day), actual hypertension (yes or no), total cholesterol/HDL-C ratio (<3.0, 3.0-5.4, ≥ 5.5), physical inactivity (yes or no) and diabetes (no or yes), # HRs and 95% CIs for ex- and occasional smokers from model including number of cigarettes smoked per day are displayed. The respective estimates in the models including tar yield, nicotine yield, tar or nicotine intake differ slightly and are almost equal.

**Table 4:** Risk of incident myocardial infarction by number of cigarettes per day (cig/day), tar and nicotine yield per cigarette (mg/cig) and tar and nicotine intake per day (mg/day) in women: Hazard ratio (HR) and 95% confidence interval (95% CI)

Smoking habits	Number of participants (N)	Number of events (N)	Age- and survey-adjusted model* HR (95% CI)	MI risk factor-adjusted model^+^ HR (95% CI)
Never-smokers^++^	2,613	49	1.00	1.00
Ex-smokers	693	15	1.95 (1.09-3.48)	1.93 (1.07-3.47)
Occasional smokers	139	2	1.56 (0.38-6.44)	1.60 (0.39-6.65)
Regular smokers				
Number of cigarettes smoked per day				
Light smokers (1 - 19 cig/day)	480	8	1.83 (0.86-3.90)	1.68 (0.79-3.57)
Heavy smokers (≥ 20 cig/day)	272	6	3.76 (1.57-8.97)	3.86 (1.57-9.50)

Tar yield per cigarette*^# ^*				
1 - 12 mg/cig	328	5	1.52 (0.60-3.85)	1.60 (0.63-4.07)
≥ 13 mg/cig	424	9	3.35 (1.62-6.94)	2.74 (1.31-5.72)
				
Nicotine yield per cigarette*^# ^*				
0.1 - 0.8 mg/cig	300	5	1.58 (0.63-3.98)	1.67 (0.66-4.24)
≥ 0.8 mg/cig	452	9	3.22 (1.55-6.68)	2.63 (1.26-5.50)

Tar intake per day*^#^*				
1 - 129 mg/day	324	4	1.27 (0.46-3.54)	1.31 (0.47-3.65)
130 - 259 mg/day	238	5	2.72 (1.08-6.88)	2.30 (0.90-5.87)
≥ 260 mg/day	190	5	5.37 (2.07-13.94)	4.67 (1.76-12.40)
				
Nicotine intake per day*^#^*				
1 - 8 mg/day	368	4	1.09 (0.39-3.03)	1.01 (0.36-2.81)
9 - 16 mg/day	235	6	3.84 (1.62-9.12)	3.88 (1.61-9.35)
≥ 17 mg/day	149	4	5.88 (2.06-16.76)	5.55 (1.88-16.35)

++ Reference category, * Model was adjusted for age (continuous) and survey (S1 or S2), model was additionally adjusted for alcohol consumption per day (0, 1-19, ≥ 20 g/day), actual hypertension (yes or no), total cholesterol/HDL-C ratio (<3.0, 3.0-5.4, ≥ 5.5), physical inactivity (yes or no) and diabetes (no or yes), # HRs and 95% CIs for ex- and occasional smokers from model including number of cigarettes smoked per day are displayed. The respective estimates in the models including tar yield, nicotine yield, tar or nicotine intake differ slightly and are almost equal.

### Risk of incident MI by tar and nicotine yield per cigarette

The MI risk increased in both sexes with rising tar or nicotine yield per cigarette per day: Compared to never-smokers, male regular smokers consuming cigarettes with ≥ 13 mg tar yield per cigarette showed a HR of 3.12 (95% CI 2.23-4.36) and with ≥ 0.8 mg nicotine yield per cigarette showed a HR of 3.01 (95% CI 2.16-4.19) in the multivariable-adjusted model. Similar results were found for women with higher HRs in the elevated tar or nicotine group.

### Risk of incident MI by tar and nicotine intake per day

Regarding the MI risk by tar intake per day estimated by a multivariable-adjusted model compared to never-smokers revealed an risk increase in male regular smokers shown by a HR of 2.24 (95% CI 1.40-3.56) for 1-129 mg/day and 3.01 (95% CI 2.08-4.36) for ≥ 260 mg/day. In female regular smokers, the corresponding associations were attenuated for low tar intake (HR 1.31, 95% CI 0.47-3.65), but more pronounced for smokers having a high tar intake (HR 4.67, 95% CI 1.76-12.40). Comparable associations with similar risk patterns were observed for nicotine intake per day.

### Risk of incident MI by number of cigarettes per day, tar and nicotine yield per cigarette

**Figure 1:**
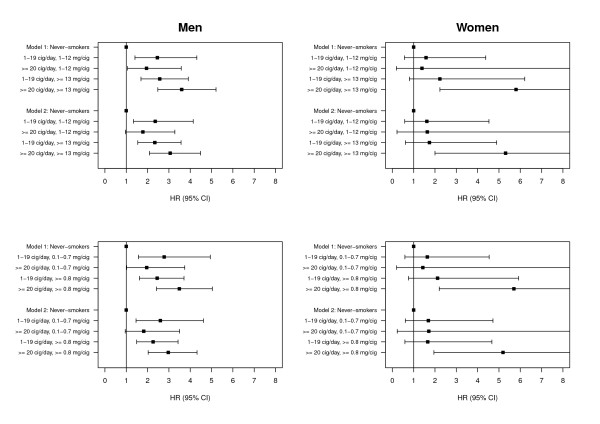
**Risk of incident myocardial infarction by number of cigarettes, tar and nicotine yield per cigarette in men and women: Hazard ratio (HR) and 95% confidence interval (95% CI)**. Model 1 was adjusted for age (continuous) and survey (S1 or S2), model 2 was additionally adjusted for alcohol consumption per day (men: 0, 1-39, ≥ 40 g/day, women: 0, 1-19, ≥ 20 g/day), actual hypertension (yes or no), total cholesterol/HDL-C ratio (<3.0, 3.0-5.4, ≥ 5.5), physical inactivity (yes or no) and diabetes (yes or no). HRs and 95% CIs for ex- and occasional smokers from model including number of cigarettes smoked per day not shown.

Finally, we analysed the joint effect of the number of cigarettes and tar or nicotine yield per cigarette by defining four categories separately for men and women (Figure 1). With respect to tar yield, the lowest category consisted of smokers with a number of ≤ 19 cigarettes smoked per day and with smoking cigarettes containing ≤ 12 mg tar yield per cigarette. Correspondingly, the highest category comprised smokers consuming 20 or more cigarettes per day and with a tar yield of 13 or more mg per cigarette. As before, never-smokers comprised the reference category.

In both sexes, the risk of an incident MI was much higher for smokers with a high number of cigarettes per day (≥ 20) and a high tar yield per cigarette (≥ 13 mg) compared to the HRs of other three categories seen by a HR of 3.06 (95% CI 2.09-4.48) in men and 5.31 (95% CI 2.00-14.07) in women in the multivariable-adjusted model. The HRs of low and high tar yield were very similar in the group with a number of ≤ 19 cigarettes per day as both HRs were around 2.35. For the joint effect of the number of cigarettes and nicotine yield per cigarette, an analogous categorization was applied with a cut-off value of 0.8 mg per cigarette and comparable results were observed.

## Discussion

The present analysis based on a large population-based sample from Southern Germany shows a substantial MI risk increase in relation to the number of cigarettes smoked per day and adds important evidence of cumulative effects of tar and nicotine intake on the risk of an incident MI in both male and female regular smokers. A dose-response-association between high daily tar and nicotine intake and MI risk in both sexes could be observed. We found that in male regular smokers even a low or medium tar and nicotine intake per day revealed a substantial risk increase as compared to never-smokers. Risks were more pronounced in medium and high intake groups in female than in male regular smokers. The findings shown in this manuscript indicate that a reduction of tar and nicotine contents in cigarettes cannot be seen as a suitable public health policy in preventing the incidence of a MI.

Tobacco smoke contains more than 4,000 distinct components during the particulates and gas phase [17]. The toxic effects of tar and nicotine are widely understood. The toxins in tar are complex and contain several major carcinogens. Nicotine, however, is related to dopamine and other neurotransmitters that might sustain smokers' addiction [18]. Nicotine concentration largely determines the way in which a cigarette is smoked [17]. Both, tar and nicotine, contribute to the increased risk for cardiovascular disease among cigarette smokers [19]. Due to the regulation and legislation of reduction in tar and nicotine emissions, a new marketing strategy, which focuses on production of new cigarette brands with low yields, has developed by the tobacco industry. The strategy is directed towards the people who have become more aware of the health effects of smoking cigarettes, and also towards smoking behaviour among regular smokers. When smokers switch to low or ultra low yield cigarettes, many of them alter their smoking behaviours to maintain their usual intake of tar and nicotine; they might increase the number of puffs per cigarette, the volume of each puff, or the duration of each puff [11].

The effects of this modified cigarette design on risk of coronary heart disease have been investigated in several studies so far and the extent of the effects have remained contradictory [8-12,20,21]. Four hospital-based case-control studies revealed that switching to lower yield cigarettes is not an effective way of reducing tobacco related morbidity from myocardial infarction [8-11]. Another case-control study with survivors of a myocardial infarction in the United Kingdom showed that even low tar cigarettes still greatly increase rates of myocardial infarction [12]. However, a large British study based on a prospective epidemiological sample of four cohorts of men has reported that smoking of reduced tar yield cigarette might lead in a modest decreased overall mortality from smoking-related diseases [21].

In the present study, gender differences have been taken into account throughout all analyses. Only few studies have investigated the relative risk of smoking on the incidence of a MI event in both sexes within the same study population, presumably in part because of an insufficient number of female regular smokers. Our results showed that female regular smokers had higher MI risks than male regular smokers in medium and high categories of daily tar and nicotine intake. This result confirmed previous studies, which showed that women are more sensitive than men to some of the risky effects of smoking [22,23]. The sex-specific differences might be explained by more pronounced harmful effects of tobacco exposure in women compared to men [23]. Moreover, a further explanation of the sex differences might be related to an interaction of sex hormones with components of the inhaled smoke. There is evidence that women who smoke are relatively deficient in oestrogen and possible biological mechanisms have been suggested [24-27].

Our study has several limitations which need to be mentioned. We could not consider effects of smoking inhalation patterns which might vary between smokers and therefore might affect tar or nicotine intake. These data were not assessed in the present study population. However, the daily cumulative concentrations of tar and nicotine in regular smokers may decrease the effects of different inhalation patterns. Moreover, we could not account for possible changes in cigarette brands and therefore possible changes in tar or nicotine intake during follow-up. However, it might be assumed that smokers choose mostly the same cigarette brand over the years and therefore that only a limited bias of the results might occur.

Our study provided additional evidence for increasing risk of incident MI among male and female regular smokers in relation to tar and nicotine intake. Although the overall distribution of tar and nicotine yield per cigarette in the present study are somewhat lower than in former studies, associations between tar and nicotine yield per cigarette and the risk of an incident MI event were clearly visible.

## Conclusions

The findings of the present study based on a large population-based sample add important evidence of the cumulative effects of tar and nicotine intake on risk of incident MI. Even low or medium yield cigarette intake increased substantially the risk of incident MI as compared to never-smokers. Therefore, reduction of tar and nicotine contents in cigarettes cannot be seen as a suitable public health policy in preventing myocardial infarction.

## Competing interests

The authors declare that they have no competing interests.

## Authors' contributions

QLZ prepared the data, performed statistical analyses of the data, contributed to the interpretation of the findings and drafted the paper. JB performed statistical analyses of the data, contributed to the interpretation of the results and writing of the manuscript and performed the final editing of the manuscript. KHL commented on the interpretation of the findings. HEW supervised the study and revisited the paper critically. CM contributed to the interpretation of the findings and revised the paper critically. AD had the idea of the study, contributed to the statistical analysis of the data and commented on the interpretation of the results. All authors read and approved the final draft of the paper.

## Pre-publication history

The pre-publication history for this paper can be accessed here:

http://www.biomedcentral.com/1471-2458/11/273/prepub
